# A qualitative feasibility and acceptability study of an acceptance and commitment-based bibliotherapy intervention for people with cancer

**DOI:** 10.1177/13591053231216017

**Published:** 2023-12-29

**Authors:** Emma Keenan, Reg Morris, Vasilis S Vasiliou, Andrew R Thompson

**Affiliations:** Cardiff and Vale University Health Board and Cardiff University

**Keywords:** acceptability, ACT, bibliotherapy, cancer, feasibility, process of change, qualitative research

## Abstract

Self-directed bibliotherapy interventions can be effective means of psychological support for individuals with cancer, yet mixed findings as to the efficacy of these interventions indicate the need for further research. We investigated the experience of individuals with cancer after using a new self-help book, based on Acceptance and Commitment Therapy (ACT). Ten participants with cancer (nine females and one male, 40–89 years old) were given access to a bibliotherapy self-help ACT-based book and participated in post-intervention semi-structured interviews. Five themes were generated from reflexive thematic analysis: (1) The value of bibliotherapy (2) Timing is important (3) Resonating with cancer experiences (4) Tools of the book (5) ACT in action. The book was found to be acceptable (self-directed, accessible, understandable content, good responsiveness to exercises) and feasible (easy to use, ACT-consistent). Although not explicitly evaluated, participants’ reports indicated defusion, present moment awareness, and consideration of values, as the ACT processes that contributed to adjustment, via helping them to regain control over their lives and become more present within the moment. Findings also indicate that the intervention may be best accessed following completion of initial medical treatment.

## Introduction

Various delivery modes have been developed, to address cancer-related psychological distress, including psychoeducation, counselling, group or individual-based psychotherapy (Grassi et al., 2017). There is good evidence that intensive forms of psychological therapy provide benefits (Chong Guan et al., 2016; Dimitrov et al., 2019). Yet, such interventions are not suitable for all, and access issues persist (Jacobsen and Jim, 2008). Some of the barriers to accessing cancer-related psychological support include resource constraints (Vaccaro et al., 2019), psychiatric symptoms adversely impacting on engagement with services (Leahy et al., 2021), stigma (Gurren et al., 2022) and individuals’ expressing concerns about the nature of the available interventions (Christy et al., 2013). Indeed, the COVID-19 pandemic has further shone a light on the need for greater variety of forms of remote delivery of psychological interventions (Wright and Caudill, 2020), and studies assessing efficacy and acceptability of such interventions are required (Capobianco et al., 2023; Nguyen et al., 2022).

Self-directed interventions requiring little to no therapist input have gained popularity (Roberts et al., 2016). One such intervention type is bibliotherapy, or the therapeutic use of books and other reading materials (Bilich et al., 2008; Howie, 1988) which can be used to address psychological and physical health concerns (Hedman et al., 2016). Among them, bibliotherapy has been used to provide support for internalizing type problems, such as depression and anxiety (Lorenzo-Luaces et al., 2023), and managing a number of long-term conditions including: chronic pain (Thorsell et al., 2011; Veillette et al., 2019), appearance related concern (Muftin and Thompson, 2013; Powell et al., 2023), distress associated with multiple sclerosis (Proctor et al., 2018) and in post-stroke management (Gladwyn-Khan and Morris, 2023).

Despite the emerging evidence base there are relatively view bibliotherapy texts aimed at supporting people with cancer, and those that exist have been subject to limited research (Brewster and McNichol, 2018). Indeed, there are only a few randomized controlled trials (RCTs) that have assessed the efficacy of cancer-focused bibliotherapy, and these have yielded mixed findings (Angell et al., 2003; Beatty et al., 2010; Carpenter et al., 2014; Körner et al., 2019). For example, Stanton et al. (2005) examined an informational workbook for individuals with breast cancer, showing improved emotional well-being, compared to those who had received the standard printed leaflets or in-person intervention. However, none of the groups reported benefits being maintained at follow-up. Likewise, in a review of ‘self-guided interventions’ for cancer patients, Ugalde et al. (2017) found only three ‘workbooks’ and the studies reporting these, had significant methodological limitations. As a result, they concluded that whilst such workbooks have promise much more research is needed before firm conclusions can be drawn. This applies to conducting further studies that will assess the effect of specific types of theoretically driven psychological intervention.

One psychological approach that is increasingly being used to provide support within clinical health settings is Acceptance and Commitment Therapy (ACT; Hayes et al., 2013). ACT is a transdiagnostic approach to psychotherapy builds upon both behaviour therapy and cognitive behavioural therapy (Hayes et al., 2013). ACT focuses on increasing psychological flexibility and the ability to engage in present moment valued driven living by cultivating six processes of change (acceptance, defusion, present-moment awareness, self-as-context, values and committed actions). ACT combines metaphors, mindfulness, experiential exercises and values-guided behavioural interventions to help individuals overcome challenges in life-threatening conditions (e.g. cancer) (Konstantinou et al., 2023; Zacharia and Karekla, 2022). Its flexible format makes it potentially scalable and effective for individuals with cancer and it has been adapted into bibliotherapy format in several other contexts (González-Fernández and Fernández-Rodríguez, 2019).

ACT’s efficacy in cancer has been reported in face-to-face therapy studies (Arch and Mitchell, 2016; Brown et al., 2020; Gentili et al., 2019; Gonzalez-Fernandez et al., 2018; Li et al., 2021a, 2021b; Low et al., 2016). Further ACT bibliotherapy’s potential is seen in other conditions, including irritable bowel syndrome (IBS) (Gillanders et al., 2017), skin and appearance-related conditions (Powell et al., 2023), multiple sclerosis (Proctor et al., 2018) and chronic pain (Veillette et al., 2019) with similar effects observed in non-clinical populations, too (Jeffcoat and Hayes, 2012; Ritzert et al., 2016). However, to our knowledge there is no existing research that has examined the acceptability of ACT for individuals with cancer delivered in the form of bibliotherapy.

Consistent with our broad objective to refine existing delivery approaches for individuals with cancer, we examined if ACT can be acceptable (e.g. elicit information about individuals’ willingness to use the intervention) and feasible (e.g. elicit information about how practical, suitable and useful the intervention is) when packaged, delivered and disseminated in a pure bibliotherapy self-help context.

## Method

### Study design

We conducted an exploratory feasibility and acceptability study, using a ‘person-based approach’ (Yardley et al., 2015) to understand the perspectives of the population who used the intervention. Employing the interpretivist methodological epistemological paradigm (Pope and Mays, 2020), we used abductive logic (Timmermans and Tavory, 2012) to enhance reflexivity, transparency and researcher-participant interdependence (Pope and Mays, 2020). Our subtle realism stance (Hammersley, 2018) aimed to identify aspects for intervention enhancement in this specific bibliotherapy-based material (e.g. reality or what we need to change in the intervention that would improve the feasibility).

### Sampling and recruitment

Convenience sampling was employed, including adults (>18 years old) with English language proficiency, diagnosed with any cancer type and self-reporting cancer-related psychological distress. We excluded individuals with an advanced cancer diagnosis (e.g. cancer that had metastasized), or those receiving palliative care as they might need more intense or personalized approaches (Rodin et al., 2020), those with significant cognitive impairment or communication difficulty, and any individuals who directly contributed to the development of the self-help book. Study participants were recruited between January and April 2021 via information sent to cancer charity organizations in the UK, such as Tenovus and Maggie’s. The selection criteria were assessed via information provided on a demographic questionnaire and via brief pre-study telephone conversations with potential participants.

#### The intervention

The intervention’s material consisted of a new book, named ‘*Living Your Life with Cancer through Acceptance and Commitment Therapy: Flying over Thunderstorms*’ (Johnson et al., 2021). The book was written by a group of senior Clinical Psychologists working with people with cancer.

The book employed ACT as a framework to cover four key areas: (1) Understanding the impact of cancer; (2) Living a meaningful life after a diagnosis of cancer; (3) Looking after yourself; (4) Moving forward. Colour illustrations are featured throughout the book to depict key ideas. Several ‘tools’ are also featured throughout the book to enhance its contents, including quotes and stories from people affected by cancer, experiential exercises, written exercises and audio exercises (accompanied by audio recordings accessible via a web link). Following permission of use by the co-authors, a proofed, pre-published version of the book was used for the study.

### Procedure

Consenting participants were either mailed a printed copy of the book or emailed an electronic version. They were asked to use the book over a period of 1 month. At the start, telephone contacts gained demographic information, addressed queries, confirmed inclusion criteria and introduced the book’s content. Digital access was confirmed, and audio files shared via the e-cloud Dropbox (https://www.dropbox.com/). Participants were encouraged to read the book fully and use the practices as much as possible. Telephone calls were also offered halfway through the intervention and at the end and used to maintain engagement and collect further data. The study received ethical approval from the South-East Wales NHS Research Ethics Committee [EC.20.07.14.6055R2A].

### Semi-structured interview guide

A semi-structured guide was created based on discussions within the research team and a review of relevant literature (e.g. Malibiran et al., 2018: see Supplemental Material S1). Acceptability definitions included traditional terms (‘social validity’ and ‘social importance’; Wolf, 1978), and alignment of the self-help material with participants’ goals. Open-ended questions covered feasibility and acceptability aspects: length, duration, style, content (exercises, material, illustration, etc), interest, usefulness, relevance, expectations met, future use and observed changes post-use.

### Data collection

Interviews were conducted via telephone following a month of assigned book work. They were recorded on an encrypted Dictaphone and lasted between 18 and 67 minutes (Mean length 42′). Following completion, interviews were transcribed verbatim and stored securely for further analyses.

### Data analysis

We employed Braun and Clarke’s (2023) suggestions for the six-phase process of the reflexive thematic analysis. The first coder (first author) listened to interviews (familiarization), read transcripts and made initial memos. NVivo version 11 aided code generation (see the Supplemental Material S2 for an example of coding sweeps in which many codes were changed, merged or removed). The coder then started generating initial themes by grouping codes relating to similar ideas or meanings. The two co-authors aided the main coder in refining codes via discussion and constant comparison with the transcripts and memos, to ensure similarities and refine codes. The coder then reviewed potential themes by considering them against the coded data, for alignment. Finally, the coder defined and named the themes subject to iterative refinement. Consistent with the iterative nature of the thematic analysis, some themes were redefined even during this final step.

### Quality assessment

We followed different approaches to demonstrate rigour in the qualitative analyses. First, the coders were guided by the 15-point checklist of criteria for good thematic analysis (Braun and Clarke, 2023). Secondly, the first author discussed all the steps of the analyses with the co-authors to reflect on the process of data analysis and explore any possible biases that may impact the analysis. Thirdly, participants were also invited to provide feedback and state whether the generated themes and subthemes reflected their words and understanding of their experiences. Further, the coder developed the main themes working across the data transcripts, mostly at a semantic level to keep proximity to participants’ experiences (inductive data-driven coding).

## Results

### Participants

Thirteen individuals responded to our request but only 10 finally consented to participate in the study. This sample size is commensurate with other qualitative explorations of bibliotherapy using thematic analysis (e.g. Gerlach and Subramanian, 2016; Malyn et al., 2020). The pool of participants had diverse socioeconomic backgrounds. Most were female, between 40 and 59 years of age, and with a higher education degree. Half of the participants were working and most of them were living with someone else. Over half had 2–3 years since cancer onset, and 6 out of 10 reported a secondary cancer diagnosis. Table 1 presents the demographics and clinical details of the study participants.

**Table 1.** Demographic and other relevant characteristics of the sample.

Demographic characteristics	*n*
Age	
40–49	3
50–59	4
60–69	2
70–79	0
80–89	1
Gender	
Female	9
Male	1
Employment	
Employed	5
Retired	5
Highest education level	
GCSE	2
A Level	1
Diploma	3
Degree	3
Postgraduate qualification	1
Cohabiting	
Y	8
N	2
Dependents	
Y	4
N	6
Physical disabilities	
Y	4
N	6
Primary cancer	
Breast	3
Kidney	1
Lung	2
Non-Hodgkin’s lymphoma	3
Skin	1
Secondary cancer present	
Y	4
N	6
Time since diagnosis	
0–1 year	2
1–2 years	0
2–3 years	5
3+ years	3
Received treatment	
Y	10
N	0
Type of treatment	
Chemotherapy	
Hormone therapy	2
Immunotherapy	4
Medication	1
Unstated	7
Radiotherapy	3
Surgery	
Stem cell therapy	1
Ablation	1
Mastectomy	2
Cancer excision	1
Currently in treatment	
Y	6
N	4
Finished treatment	
Y	2
N	8
Psychological problems before cancer	
Y	10
N	0
Psychological support received	
Y	6
N	4

### Findings

In the Supplemental Material S3, we present the five themes, subthemes and the number of participants who contributed to generating these themes. We provide direct codes from participants, using pseudonyms, to allow others to judge whether the interpretation we provide to support the study findings is adequately grounded by the data.

## Theme 1: The value of bibliotherapy

### Accessibility

Most participants found bibliotherapy accessible. Accessibility was discussed in several ways, including how convenient the self-help book was to use:

*I will buy a hard copy of the book when it comes out because I just think it was a great book to have. . . it’s a handbag book then isn’t it. . . it might be something that I can just carry around with you or have on the bedside a bit more conveniently (Penny)*

Participants also mentioned how easy it was for them to use the book when other sources of support were unavailable, such as professional help or close personal relationships.

*. . .you are isolated, so you are going back to a book, and you are finding it as company, in a way. Because you are reading something which is helping, you are almost talking it over with yourself and instead of seeing other people, you are going through the book, and it is almost like a friend (Elizabeth)*

The book was also found to be accessible in terms of participants’ ability to use it at any time. This was of particular importance to some participants who experienced symptoms such as pain and fatigue that impacted on their ability to consistently engage with other sources of psychological support.

*You have really good and really bad days, and during treatment you have some horrible days worse than others that you’re just so ill, but you can’t predict it. That horrible day may be on the day that you’ve got this appointment that was booked three months ago because you’re on a waiting list to see a psychologist. You don’t want to miss that appointment, but you feel so crap you can’t get out of bed, and you’re not really going to benefit out of that session because you’re probably not going to listen or engage as much because you feel unwell. Whereas if you’re feeling like that and you can pick up a book and think, ‘Do you know what? This section will help me today. I’ll read this and see if it can help me’. (Vanessa)*

### Usability

Participants had positive comments about the structure of the book. The ability to maintain focus on the book was particularly significant to some participants who highlighted how cognitive difficulties (e.g. fatigue, lack of concentration) can interfere with intervention engagement.

*The pages are big and it’s easy to read. . . I liked the way it was set out with the bullet points and the little drawings and things like that, it kept your attention because it wasn’t pages and pages of text which I did find helpful. Because at any time your mind tends to wander off from things but especially when you’ve got things going on (Tina)*

Many participants discussed the benefit of being able to easily ‘*dip in and out*’ of the book, reading sections that feel the most relevant in that moment.

*This is the kind of book that you can just pick up and because of the way it’s laid out, you can just go to a relevant chapter, and I think it’s done in a way that would enable you to tackle things that are bothering you at that time. (Penny)*

Many participants also discussed the benefit of the book being an intervention you can return to during different phases of diagnosis and treatment. This was felt to create a person-centred form of support and promote longevity of the intervention, which resonated with participants’ experiences of cancer being a ‘*journey*’.

*When I thought about it, using the book. . . I thought a trellis is a great idea because you’re the plant obviously growing but you just need that little bit of support and then once you’re on one bit of support, sometimes you’ll need another different bit of support so you’re moving around and up this trellis, onwards, outwards, sideways, every direction depending on what sort of a plant you are. . . a ladder is one thing, but a ladder sounds like you’ve got a direction, you know you’ve got to go somewhere, a trellis is just you could be going in any direction it doesn’t matter, you know there’s no necessary right or wrong way to go it’s just a support whichever sort of plant you are. (Hazel)*

## Theme 2: Timing is important

Participants reported that receiving a bibliotherapy intervention straight after diagnosis may be too overwhelming. Cheryl indicated she could not have engaged with bibliotherapy following diagnosis, as she was ‘*still reeling with it all*’ and Eve suggested ‘*there’s just so much that you can’t take it on board if you’re newly diagnosed*’.

*I think just after diagnosis your head is, well, for me my head was just so scrambled. . . if I’d walked past [the book] when I was in the treatment centre or something, I might have picked it up, but I think I probably would have not got so much from it as I have now, having sort of two years between diagnosis and now. (Tina)*

Many individuals recommended that an optimal time frame for the intervention provision would be after treatment.

*I think right at the beginning would have been too soon. . .Basically, you’re in denial first. . . then you start thinking about the long term. In your head when everybody’s sleeping and you’re awake, you start thinking about it. Then when you start your treatment, it becomes a bit real. When I started getting some of my side effects, you tend to spend a lot of time in bed or poorly, and that’s when you need something to read that’s actually going to help you. When I was in chemotherapy, really ill in bed and I was up in the middle of the night being ill, that would have been a good book to say, ‘Look, this is what you need, this will help you’. (Vanessa)*

## Theme 3: Resonating with cancer experiences

### Recognizing elements of own cancer journey

All participants resonated with some of the content in the book. For some, this involved relating to key ideas or metaphors in the resource.

*The book caused me to think. . .for example the concept of wearing a mask, which is what I’ve been doing, because I’ve been stressed out and not in in good spirits for about three years, because all this started three years ago and I have just pretended. I’ve got three sons who live locally, and I’ve just pretended that everything is fine and I’m fine and there are no issues, but it’s just completely untrue. . . so when the book talked about wearing a mask, that resonated with me. (John)*

For others, relatable cancer experiences were found within the quotes from those with experience of living with cancer.

*[quotes] like “I have gone from being a well person who feels in control of my life and my future, to facing the unknown with no road map” - that was a really good quote from someone [mentioned in the book], that resonated with me. (Nicola)*

### Normalizing experiences

Relatable content within the book was reported to normalize cancer experiences for many participants, including personal responses to diagnosis and difficulties related to the reactions of others.

*It reinforces that sometimes the slight annoyance I feel when people say, “Oh, you’ve done so well, time to move on” and all this sort of stuff, sometimes I get annoyed about that because I think, “you really don’t get this”. So, to understand that all of those feelings are completely normal. . . and even if you know in your heart of hearts that what you’re feeling is normal and actually they are coping mechanisms, just to dip in and read that and reinforce how you’re feeling I think is really, really good. (Penny)*

### Validating experiences

Participants reported that recognizing aspects of their own cancer journey in the book, such as the use of certain coping strategies, helped to validate their cancer experiences. This seemed to increase motivation to continue using the intervention.

*And that’s basically what you were saying in your book and so it’s so lovely that it all ties up and the things that I’ve been trying to do over the past two years have really kicked in and made a difference. (Eve)*

## Theme 4: Tools of the book

### Interactivity

Most participants identified a feature of the book that was meaningful and helpful to them for example, illustrations, exercises, quotes.

*I liked some of the graphics, some of the illustrations, writing down your own distractions, solving problems – all those sorts of things I thought was really useful because it felt like a workbook then, that you could add something to it that was important to you. (Nicola)*

### Audio exercises

Four participants did not engage with the audio content provided via link within the book, although two of these participants did report that they engaged with the audio exercises via reading the accompanying scripts. Reasons for not engaging in audio exercises included lack of time, accessibility of audio files and personal preference.

*I did only listen to a couple of them, but I found it easier just to read it actually, for me, and to sort of be able to go back and read it a bit again rather than having it as audio. (Tina)*

The six participants who did engage with the provided audio content indicated that audio exercises facilitated engagement with several core ACT processes, including being in the present moment, acceptance and cognitive defusion.

*You had the book but then you had the audio exercises that went along with it which really did add to it. . .the three-stage breathing which I thought was good as well because you find it calms you down as well, it sort of brings you back into the moment, it focuses you a little bit more, which I found was great because your mind just does tend to wonder and you are thinking about the next treatment or the next appointment. (Elizabeth)*

### Written exercises

Six out of ten participants reported that they engaged with written exercises. Reasons for not responding to the written material in the book included time, personal preference and having completed similar activities in the past. A number of those who did engage with the material said that it was helpful and ACT processes of change facilitated their engagement. For example, Nicola reported ‘*the values list I really enjoyed doing. . . there were things in there like learning, I had forgotten how much I like to learn*’. Tina also completed several written activities and described how one helped her with the process of defusing her thoughts:

*I normally sort of shy away from things like that if it had been in some other subject but, you know, having tried one or two [written exercises] at the beginning. . . especially that one I said where I wrote the things before about noticing about what you were thinking and I realised that really, really did work for me. (Tina)*

Some participants reported that they revisited written tasks after their completion to reflect on the material at hand. For example, Elizabeth said ‘*it was like I wrote it down and then the following day I went back to see what I had written – oh yes, I get it now!*’

## Theme 5: ACT in action

### Observe and notice

Several participants reported a better management of cancer-related distress using the practice of distancing (e.g. separating from thoughts) to observe their thoughts, instead of having them drive their behaviours.

*I liked the way it worked through the fact that you can regain control of some of your feelings and, not just regain control necessarily, but you can observe and note and notice improvements and see what’s going on and check in with yourself. (Hazel)*

Interestingly, participants’ experience with distancing was perceived as an activity that facilitated better control over the impact cancer-related thoughts can have on their actions.

*I think just the initial idea of looking at your thoughts from a slightly detached space. . . I think I wrote down a thing of “I noticed that I’m thinking about a certain thing” . . . doing little things like that actually did really work for me. I did say out loud “the thing that was worrying me” and then I realised that when you then put in those other words before it. . .it actually does take away the power of it. . .you realise that it’s something perhaps you can have more say over rather than it just controlling you. . . that has made a real conscious thing that I can think about that in a different way now. (Tina)*

### Regaining control of my values

Engagement with the activities pertaining to values, allowed several participants to regain a sense of control over their priorities. For instance, many shared their experiences of having to make sacrifices, such as giving up their jobs, adjusting their roles within their families and friendship circles, and reorganizing their daily routines to accommodate medical appointments. Engaging with content in the book relating to values seemed to help participants reflect on these changes, revaluate what mattered to them and set specific committed actions towards value-based goals.

*The book has helped me thinking about how I take control and what I do, and that is mainly my relationship with others, with the oncologist, with people who maybe have suggestions for you, thoughts for you, ideas, you should do this, do that. (Nicola)*

### Living mindfully

Several participants discussed positive outcomes from engaging with mindfulness exercises, including feeling more relaxed, improved sleep, connecting with emotions and increased capacity to replace automatic reactions with more conscious responses.

*The most I really did find really helpful was the mindfulness, because it does let you put things out of your mind. . .maybe I’ve just been running on adrenalin sort of thing, and I’ve never really sort of slowed down. . . I have taken away the mindfulness. (Valerie)*

Some participants described becoming very busy after finding out they had cancer. Being present seem to have helped participants stay grounded.

*The breathing, so it talks about keeping still, pushing your feet on the floor and breathing. And, just thinking about where you are now. That sort of thing just brings you back, brings you back to now. Don’t let your mind run away too far away (Nicola)*

Additionally, some participants went on to incorporate mindfulness exercises into their daily routines.

*I have noticed that I do the mindful walking a bit more. . . I used to go out and whenever I went for a walk, I used to put headphones on. I’ve stopped doing that now and I just go for a walk and listen to my footsteps, listen to the noises around me and just try and switch off. (Elizabeth)*

## Discussion

Bibliotherapy is a promising approach for delivering psychological interventions, emphasizing self-help with minimal or no therapist contact. Yet very few self-help books have been subject to any form of testing to ascertain cancer patients’ views. We evaluated the acceptability and feasibility of a new commercially available ACT-based bibliotherapy intervention (Johnson et al., 2021). Five themes were generated including (1) The value of bibliotherapy (2) Timing is important (3) Resonating with cancer experiences (4) Tools of the book (5) ACT in action. Analysis showed that these themes reflected aspects of ACT’s feasibility and acceptability when delivered in a form of bibliotherapy.

Acceptability findings show the intervention’s accessibility and self-directed nature. This is in alignment with the limited literature on distress alleviation for individuals with cancer (Semple et al., 2006, 2009), and the results are consistent with evidence for interventions focusing on mental health contexts (Andrews et al., 2010; Jones et al., 2021; Levis et al., 2022; Li et al., 2012; Rodin et al., 2020) or other physical conditions (Andersson et al., 2014). ACT’s acceptability was also evident in response to feedback, provided on the book tools such as the quotes, illustrations, audio exercises and the written exercises used. These all appeared to have enhanced understanding and provided a person-centred experience. This aligns with ACT’s process-based approach that targets functionally important processes of change, leading to desired long-term positive outcomes (Hayes et al., 2020). To this end, values and mindfulness may be considered key processes of changes in the psychosocial support of individuals with cancer (Hayes et al., 2020), indicating ‘foci’ for treatment delivery.

In terms of feasibility, findings showed that participants found the book easy to use. This is a pragmatic feasibility parameter that gives credits to bibliotherapy as being potentially effective means of delivery for cancer-related population who frequently experience fatigue, pain and cognitive difficulties (Martin et al., 2012). These difficulties can indeed limit engagement with bibliotherapy interventions which usually require good attention span capacity. Our findings demonstrated individuals’ engagement with the intervention, indicating the scalability of ACT, delivered in different forms.

An unexpected finding concerned the timing of bibliotherapy intervention. Our findings indicated that receiving bibliotherapy right after diagnosis might be overwhelming due to intense cancer-related emotions (e.g. sadness, anger, anxiety, etc.; Cincotta, 2004). Participants indicated delayed intervention provision as more suitable due to potential engagement difficulties or reduced benefits immediately post-diagnosis. Our study aligns with existing evidence (Angell et al., 2003; Brebach et al., 2016) suggesting that following medical treatment might be the optimal time for providing bibliotherapy to this population. The benefit of providing the intervention following completion of acute treatment maybe that this enables patients to continue to use their existing coping resources. Also, there may be some time for the impact of the disease to be established prior to psychological intervention being instigated (Holland and Reznik, 2005).

The study had the following limitations. Firstly, the qualitative nature of the data collection did not allow us to quantify measurements of feasibility (e.g. time spent in the intervention) and acceptability (e.g. frequency of material used). Secondly, there was a noticeable lack of diversity among participants. Despite our efforts to recruit individuals from different centres, all but one of the participants were female, and the entire sample was aged 40 years or over, indicating a lack of heterogeneity within the sample. It is also important to note that participants consisted mostly of self-selected individuals with less advanced cancer and higher educational attainment. Thus, the generalizability of our findings our limited. Furthermore, recruitment took place during the COVID-19 pandemic and therefore it is possible participants may have been even more motivated to look for alternative means of support during this time when access to alternative, face-to-face intervention may have been less readily available. Thirdly, all participants reported previous experience of cancer-related psychological difficulties, and over half had accessed to some sort of psychological support for this. It is unclear how previous psychological support may have impacted participants’ experiences of the current intervention. A fourth limitation of the study is the small sample size and follow-up period of 1-month after assigned book work. It would be beneficial for further studies to include larger sample sizes and longer follow-up periods. Finally, the authors’ beliefs and assumptions during data analysis may have inflated possible areas of bias, including the main researchers’ prior understanding of bibliotherapy, cancer treatment experiences, and the authors experience of the use of the ACT approach. Arguably, these factors might have shaped the findings in some way (Braun and Clarke, 2023). It is with this in mind, we employed the subtle realism (Hammersley, 2018) approach to attempt to represent individuals’ experiences rather than to attempt to attain a real ‘truth’ (Pope and Mays, 2020).

Feasibility studies aim to hasten ‘real world’ implementation of evidence-based interventions, carrying theoretical, research and practical implications. Participants found ACT-based bibliotherapy components compatible with their cancer experiences, fostering normalization and validation that research suggests reduces distress (Clarke et al., 2021; O’Hea et al., 2016). Notably, our findings indicate time-delivery issues. Professionals can recommend bibliotherapy around the time treatment ends, either alone or alongside mild distress or while waiting for professional support (Kangas and Gross, 2020; McDonnell et al., 2022; Macía et al., 2022). There is a need for further investigation of the combination of delivery mode and psychological approaches (Caruso and Breitbart, 2020) to support the present study findings.

In conclusion, participants found the ACT-based self-help bibliotherapy as an acceptable and feasible form of psychological intervention. Although not explicitly or quantitively evaluated, participants’ accounts indicated that the intervention, overall, worked via its treatment mechanism with defusion, present moment awareness and values to contribute to better coping with cancer adjustments, including individuals’ sense of regaining control over their lives and being more present. There is merit in further understanding how bibliotherapy, and particularly the interaction between this mode of delivery and ACT’s processes of change, may benefit the cancer population. This will ultimately lead to more personalized, modularized psychological support interventions.

## Supplemental Material

sj-docx-1-hpq-10.1177_13591053231216017 – Supplemental material for A qualitative feasibility and acceptability study of an acceptance and commitment-based bibliotherapy intervention for people with cancer Supplemental material, sj-docx-1-hpq-10.1177_13591053231216017 for A qualitative feasibility and acceptability study of an acceptance and commitment-based bibliotherapy intervention for people with cancer by Emma Keenan, Reg Morris, Vasilis S Vasiliou and Andrew R Thompson in Journal of Health Psychology
