# Supplementary material for: A qualitative feasibility and acceptability study of an acceptance and commitment-based bibliotherapy intervention for people with cancer
Source: J Health Psychol. 2023 Dec 29;29(5):410–24. doi: 10.1177/13591053231216017 (PMC11005316; doi:10.1177/13591053231216017)
Supplement: sj-docx-1-hpq-10.1177_13591053231216017 – Supplemental material for A qualitative feasibility and acceptability study of an acceptance and commitment-based bibliotherapy intervention for people with cancer [file sj-docx-1-hpq-10.1177_13591053231216017.docx]

Supplementary Material S1: **Interview Schedule**

1. Could you briefly tell me about your experience of living with cancer before using the book?
2. What interested you about the book?
3. Before using the book, did you have any expectations of it? If so, could you tell me about these?
4. How did you use the book? (Prompts: Did you read the book from beginning to end or read different sections at a time? Did you leave any sections out? Did you try the written and audio exercises?)
5. Could you tell me about your experience of using the book? (Prompts: How did using the book make you feel? Did any parts of the book stand out for you? Did any parts feel more of less relevant? What was your experience of the written exercises? What was your experience of the audio exercises?
6. How did your experience of the book compare to your expectations of it?
7. How would you describe the book to someone?
8. Could you tell me about any changes that you have noticed or made since using the book (if any)? (Prompts: thoughts, feelings, behaviours, ways of coping, lifestyle changes)
9. How do you feel about using the book again in the future?
10. Are there any other comments you would like to make about the book?

**Demographic Questionnaire**

**Participant Questionnaire**

*The information provided for this questionnaire will be used anonymously in the study. Please answer as many questions as possible. However, you do not have to*

*answer anything that you don’t want to. Thank you.*

**Today’s date:** ____/____/______          **Participant Number [office use]:** _______

**Age:** ________

**Gender:** Male □ Female □ Prefer to self-describe as__________________ □

**Are you currently in employment?** Yes / No **Are you retired?** Yes / No

**Current / previous work:**

___________________________________________________________________

___________________________________________________________________

**At what age did you leave education?** ___________________________________

**Highest level of qualification obtained?** _________________________________

**Do you live by yourself?** Yes / No **Number of dependents** _________

**Do you have any physical disabilities:** Yes / No

**When were you diagnosed with cancer?**_________________________________

**What type of cancer were you diagnosed with?** ___________________________

**Have you been diagnosed with more than one type of cancer?** Yes / No

**If yes, what other types of cancer have you been diagnosed with?**

___________________________________________________________________

**Have you had treatment for your cancer?** Yes / No

**If yes, what treatment have you had?**____________________________________

**Are you currently in treatment?** Yes / No

**Have you finished treatment?** Yes / No

**Have you been affected by psychological problems such as anxiety or depression since you were diagnosed with cancer?** Yes / No

**If yes, please tell us how you were affected:**

___________________________________________________________________

___________________________________________________________________

___________________________________________________________________

**Have you received any treatment/support for this?** Yes / No

**If yes, please tell us about the treatment/support you received:**

___________________________________________________________________

___________________________________________________________________

___________________________________________________________________

*Thank you for completing this questionnaire*

Supplementary Material S2: **Sample from the analytic stages followed**


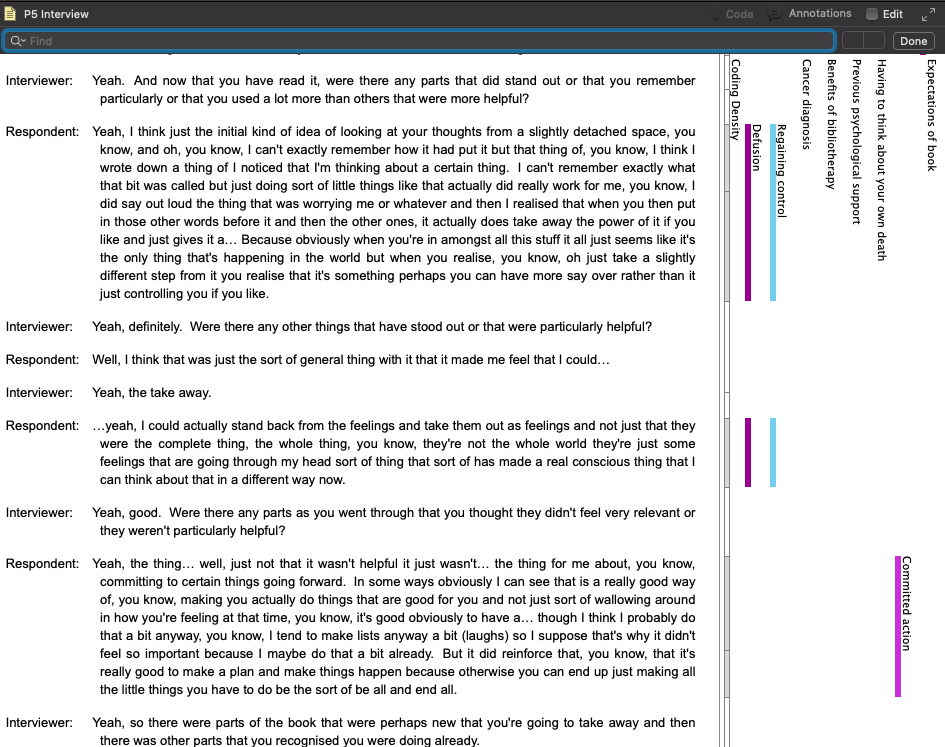


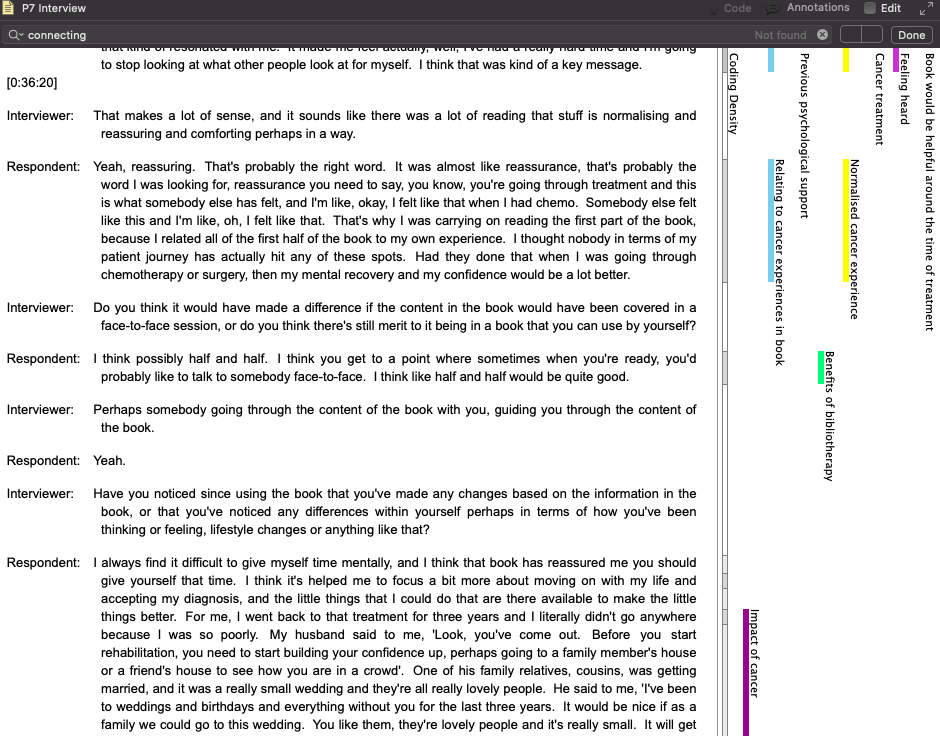


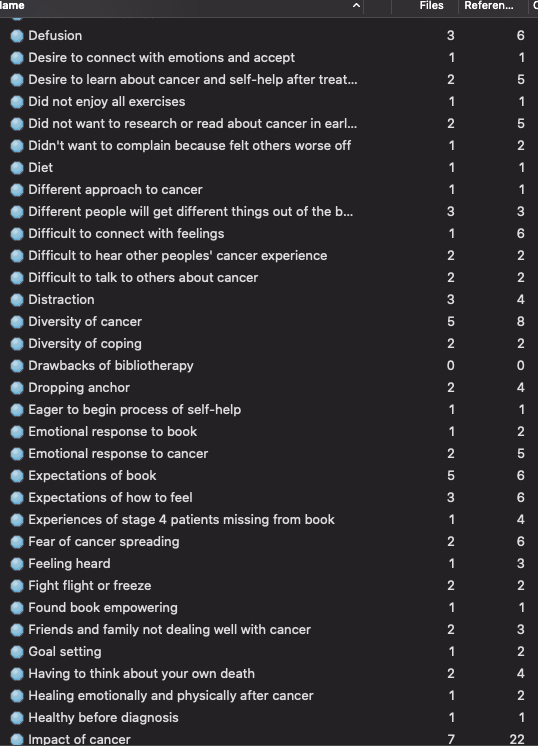


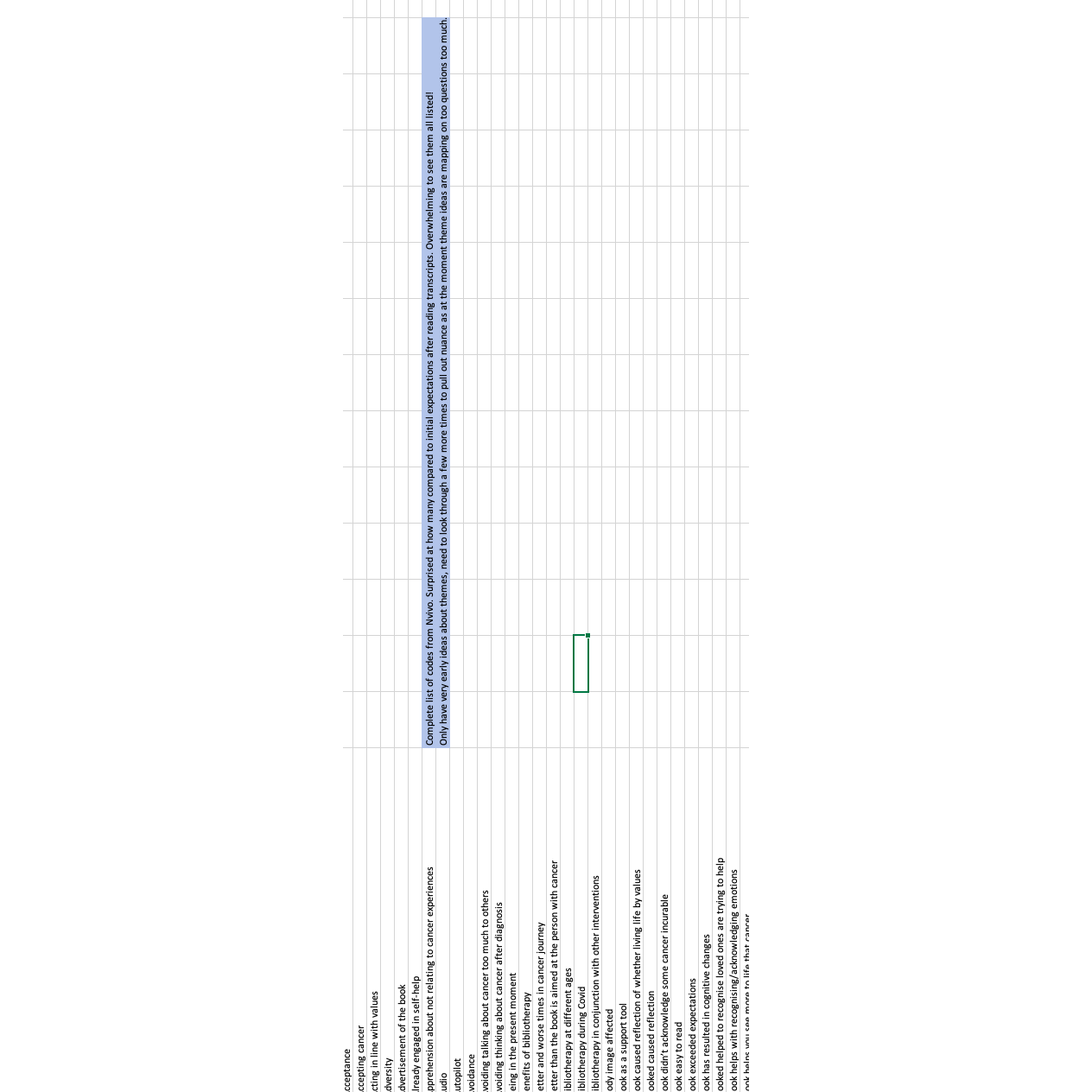


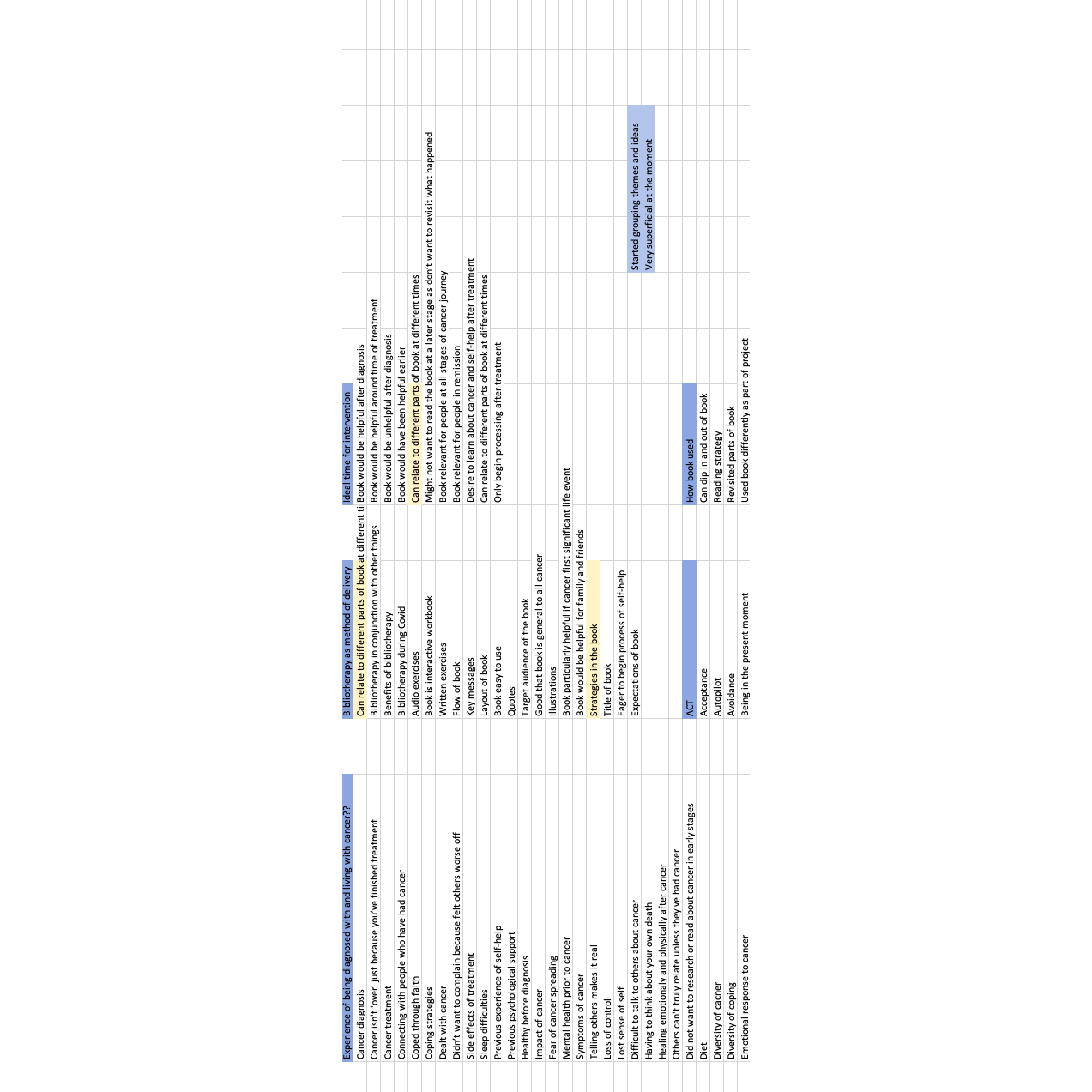


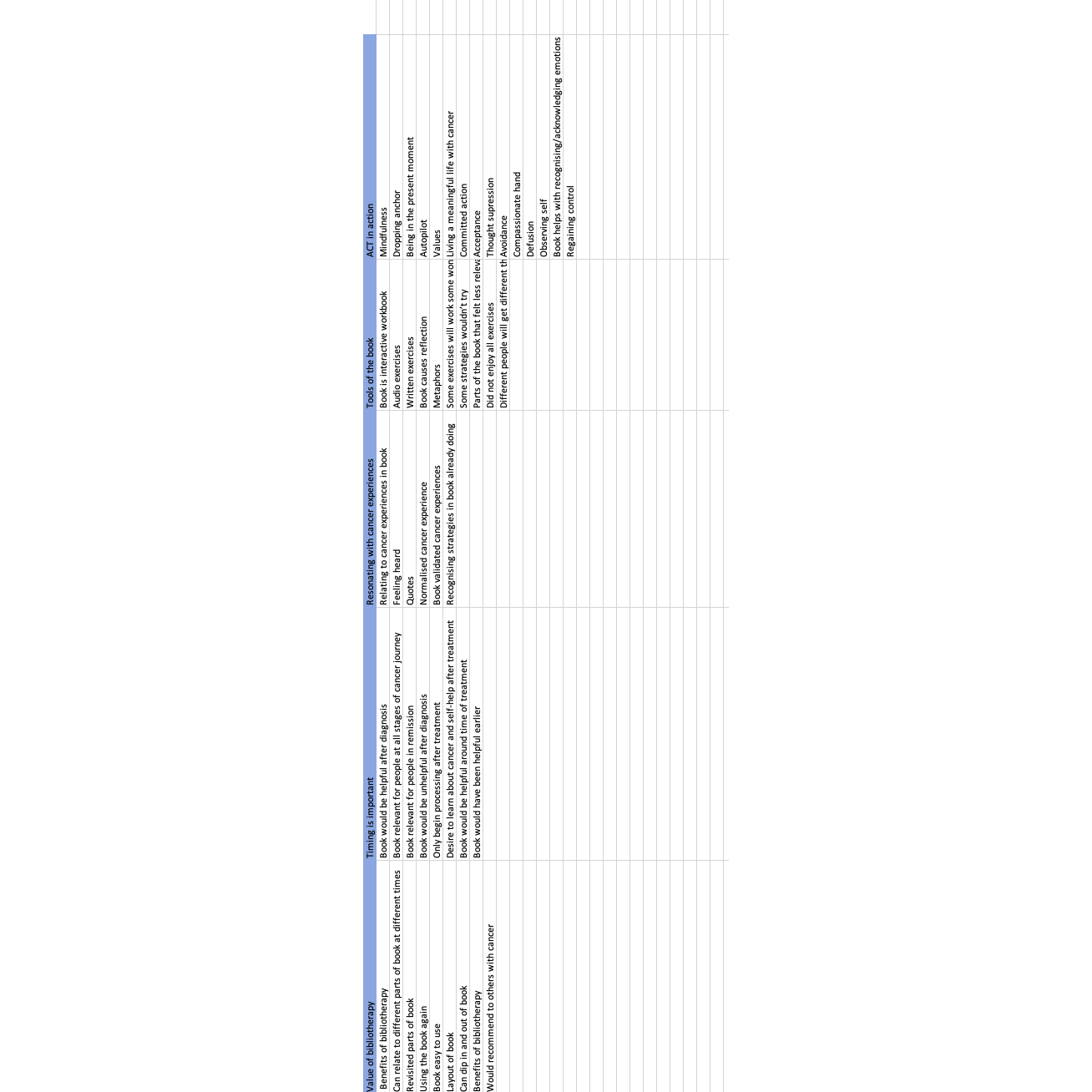


Supplementary Material S3: **Themes, subthemes, and contributors**

| Themes | Subthemes | Contributors |
| --- | --- | --- |
| Theme 1: The value of bibliotherapy | - Accessibility - Usability | 9 |
| Theme 2: Timing is important |  | 9 |
| Theme 3: Resonating with cancer experiences | - Recognising elements of own   cancer journey   - Normalizing experiences - Validating experiences | 10 |
| Theme 4: Tools of the book | - Interactivity - Audio exercises - Written exercises | 10 |
| Theme 5: ACT in action | - Observe and Notice - Regaining control of my Values - Being present | 9 |

Note: Thematic analysis and its ability to identify nuanced patterns across meaning-rich data can be useful in facilitating a closer exploration of processes of change that may be occurring for service users because of engagement with interventions. Analysis was inductive and carried out at a semantic level to reflect the explicit content of participants’ accounts.
